# A Narrative Review on the Viability of Osteopathic Manipulative Medicine in Treating Irritable Bowel Syndrome With Constipation (IBS-C)

**DOI:** 10.7759/cureus.54180

**Published:** 2024-02-14

**Authors:** Mahi Basra, Hemangi Patel, Alison Stern-Harbutte, David Lee, Randal K Gregg, Holly B Waters, Anna K Potter

**Affiliations:** 1 Foundational Sciences, Nova Southeastern University Dr. Kiran C. Patel College of Osteopathic Medicine, Clearwater, USA; 2 Research, Nova Southeastern University Dr. Kiran C. Patel College of Osteopathic Medicine, Clearwater, USA; 3 Osteopathic Medicine, Nova Southeastern University Dr. Kiran C. Patel College of Osteopathic Medicine, Clearwater, USA

**Keywords:** gut microbiome, pelvic floor dysfunction, inflammatory bowel disease, autonomic nervous system dysfunction, ibs (irritable bowel syndrome)

## Abstract

Irritable bowel syndrome (IBS) is a functional gastrointestinal disorder characterized by chronic abdominal pain and alterations in bowel habits, with global prevalence. The etiology of the disease is likely multifactorial; however, autonomic nervous system (ANS) dysfunction and immune-mediated inflammation may contribute the most to the hallmark symptoms of abdominal pain and altered motility of the gut. Current pharmacological therapies operate to modulate intestinal transit, alter the composition of the gut flora and control pain. Non-pharmacological approaches include dietary changes, increased physical activity, or fecal microbiota transplants. None of these therapies can modulate ANS dysfunction or impact the underlying inflammation that is likely perpetuating the symptoms of IBS.

Osteopathic Manipulative Medicine (OMM) is a clinical approach focused on physical manipulation of the body’s soft tissues to correct somatic dysfunctions. OMM can directly target the pathophysiology of IBS through many approaches such as ANS modulation and lymphatic techniques to modify the inflammatory mechanisms within the body. Particular OMM techniques of use are lymphatic manipulation, myofascial release, sympathetic ganglia treatment, sacral rocking, counterstrain, and viscerosomatic treatment. The aim of this study is to identify OMM treatments that can be used to potentially reduce the inflammation and ANS dysfunction associated with IBS symptoms, thereby providing a new non-pharmacological targeted approach for treating the disease.

## Introduction and background

**Figure 1:**
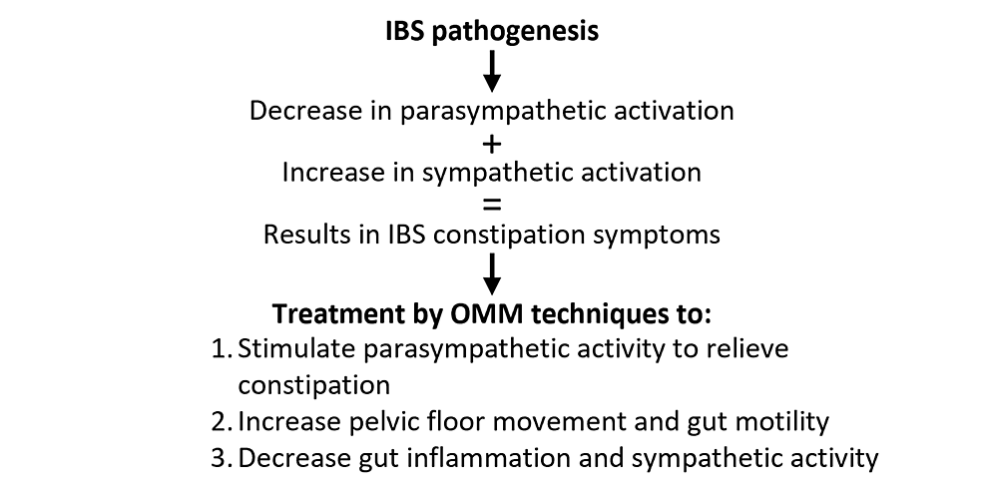
Schematic of irritable bowel syndrome pathogenesis followed by potential IBS treatment using osteopathic manipulation medicine. Alteration to sympathetic and parasympathetic activity and gastrointestinal movement are hypothesized as suitable treatment options for patients who suffer from IBS. Original artwork by authors.

Irritable bowel syndrome (IBS) is a chronic, inflammatory gastrointestinal disorder that affects the large intestine [1]. IBS affects between 5-10% of individuals in the world and 4-5 million individuals in the United States [2]. Symptoms of IBS range from abdominal pain, diarrhea, cramping, bloating, gas, and constipation [1]. Due to the chronic nature of this disease, the pathogenesis and exacerbating factors have been heavily investigated. The pathogenesis has been attributed to four main components: autonomic nervous system (ANS) dysfunction, immune-mediated inflammation, disordered gut-brain axis (GBA), and microbiome dysbiosis [2]. An increase in pro-inflammatory cytokines, alteration in mucosal mechanisms, and expression in receptors play a vital role in IBS pathogenicity [3]. The gut microbiome and GBA have also been shown to modulate neuroinflammation and inflammatory processes (Figure 1).

The Rome III classification categorizes irritable bowel syndrome (IBS) into four subtypes. The focus of this paper is IBS with constipation (IBS-C), which presents with abdominal pain. Pelvic Floor Dysfunction (PFD) is defined as impairment and spasms in pelvic floor musculature affecting the lower gastrointestinal tract [4]. Constipation, increased stool straining, and impaired defecation can all result from PFD. In IBS-C, patients predominantly suffer from constipation and increased stool straining, which can lead to overactive pelvic floor muscles and subsequent dysfunction [5].

The gut microbiome is composed of trillions of microorganisms inside the gastrointestinal tract of humans [1]. Together, these microorganisms have an impact on numerous different biological processes through the GBA [1]. The GBA has been described as a bidirectional communication pathway between the central nervous system (CNS) and the gut [1]. Communication between the CNS and gut involves multiple systems including the hypothalamic-pituitary-adrenal (HPA) axis, enteric nervous system (ENS), and immune system [1]. Microbiota within the gut play a role in this communication system and the composition of the microbiome can have a direct impact on the various systems involved in the GBA [1]. Dysbiosis describes imbalances within the gut microbiome leading to disruptions within the GBA and causing alterations in gut motility, secretion, and sensibility [1]. These factors have been proposed to play a role in functional GI disorders such as IBS [1].

Osteopathic medicine is a philosophy, science and art that emphasizes the concept of the body as a whole unit. Osteopathic philosophy is applied in structural diagnosis while osteopathic manipulative treatment (OMT) is used to treat patients. The tenets of osteopathic medicine focus on four main points. First, the body is a unit and that unit is made up of the body, mind, and spirit. Second, the body is capable of self-regulation, self-healing, and health maintenance. Third, structure and function are reciprocally interrelated. Fourth, rational treatment is based on the principles of body unity, self-regulation, and the interrelationship of structure and function. These four tenets set a precedent for osteopathic physicians and how they should approach the treatment of their patients [6]. 

The heterogeneous pathology of IBS presents a challenging obstacle to overcome due to several factors involved including stress, anxiety, diet, changes in the GBA, gut microbiota, gastrointestinal impairment, and genetic factors [1]. Thus, osteopathic medicine and its holistic treatment plan present an intriguing and overlooked approach to the management of IBS. Current non-pharmacological treatments of IBS focus on diet (low-FODMAP and high fiber), physical activity, fecal microbiota transplants, and psychological treatments. This review aims to explore osteopathic manipulative medicine (OMM) techniques as a non-pharmacological approach to IBS treatment.

Underlying pathology of irritable bowel syndrome

Imbalance of the autonomic nervous system (ANS) and enteric nervous system (ENS), PFD, and mucosal inflammation have been explored as possible pathological causes of IBS-C. Dysfunction of these systems is thought to contribute heavily to the hallmark symptoms of IBS-C: abdominal pain and constipation.

Imbalance of the Autonomic Nervous System

The ANS monitors and controls several physiologic processes including blood pressure, body temperature, digestion, energy balance, excretion of wastes, fluid volume, and glucose homeostasis [7]. Through the GBA, the ANS has a direct impact on gut motility, visceral sensitivity, and immune response of the gastrointestinal tract [8]. Activation of the sympathetic autonomic nervous system (SANS) promotes a predominantly inhibitory effect over gastrointestinal tone and a tonic inhibitory effect over gastrointestinal secretions [7]. The parasympathetic autonomic nervous system (PANS) opposes the SANS by promoting excitatory control over gastrointestinal tone and motility [9]. 

The PANS modulates the activity of the gastrointestinal tract through the vagus nerve (VN) and pelvic splanchnic nerves (PSN). The VN is a vital component of the PANS, which helps to control mood, immune response, digestion, blood pressure, and heart rate. Originating in the brainstem and traveling to the abdomen, the VN carries signals between the digestive system and the brain. It controls contractions of smooth muscles in the intestines and gland secretion. Preganglionic efferent fibers come from the dorsal motor nucleus located in the medulla to innervate the lamina propria and muscularis externa layers in the gut. Vagal afferents include mechanoreceptors in the mucosa, chemoreceptors, and esophageal tension receptors among other sensory receptors. These send information to the nucleus tractus solitarius (NTS). NTS sends the sensory information to areas in the central nervous system such as the amygdala and thalamus [10]. The PSN consists of nerve bundles from S2-S4, which are responsible for the parasympathetic activity and pain perception of the bladder, left colon, sigmoid colon, and rectum [11]. 

Recent studies have concluded that ANS dysfunction is thought to heavily contribute to the hallmark symptoms of IBS-C [12-19]. Studies focused on IBS-C patients have shown that compared to healthy controls, IBS-C patients suffer from increased sympathetic response and decreased parasympathetic response [12,13,17,18]. The reduced activity of the parasympathetic component of the ANS has been correlated to the decreased vagal tone measured in IBS-C patients [10]. 

Autonomic activity testing in patients with IBS-C has revealed documented changes in both sympathetic and parasympathetic activity, as evidenced by blood flow measurements in their fingertips [12]. Along with autonomic testing, increased catecholamine levels have been observed, further reinforcing the finding of increased sympathetic tone in IBS-C patients. Vagal activity provides a protective function to the intestinal epithelium and helps manage immune reactions in the gut. Decreased vagal activity increases intestinal epithelial permeability further promoting inflammation and chronic disease [12].

The Enteric Nervous System

Known as the “brain within the gut”, the ENS can control motility, blood flow, and immune response within the gastrointestinal system [8]. Derived from neural crest cells, the ENS is made up of the submucosal plexus and the myenteric plexus [10]. Embedded in the wall of the entire gastrointestinal tract, these two plexuses can stimulate peristaltic movement independent of the CNS [8]. Although the ENS primarily works alone, it is still considered part of the ANS. Communication between the ENS and the ANS occurs through the VN, PSN, and the paravertebral sympathetic chain [20]. Neurotransmitters such as serotonin and acetylcholine are used as chemical signals to help the ENS and ANS control the digestive system [10]. 

Pelvic Floor Dysfunction

PFD encompasses a broad range of anatomical changes in pelvic floor musculature. PFD is common in disorders of the genitourinary tract and lower gastrointestinal tract. The pelvic floor is composed of muscles and their attachments creating a pelvic diaphragm that spans the pelvic outlet. The pelvic floor muscles are collectively called the levator ani; the muscles composing this group are pubococcygeus, iliococcygeus, and puborectalis. The puborectalis muscle acts as a muscular sling that surrounds the anorectal junction. Superficial to the pelvic floor musculature lies the external anal sphincter.

Dysfunction of the pelvic floor can arise from hypertonic muscles, hypotonic muscles, and or disharmony of the pelvic floor muscles. Disturbances to the pelvic floor can impact the genitourinary and gastrointestinal tract, leading to urinary urgency and incontinence, fecal incontinence, and pelvic organ prolapse. The common presentation of colorectal disturbances in PFD is the inability to evacuate the lower colon and constipation. In IBS-C patients it is postulated the hypertonic pelvic floor muscles cannot relax and can lead to stool straining and fecal incontinence [4].

Symptoms of IBS-C, such as constipation and bowel straining can further exacerbate PFD. Women with IBS have reported experiencing fecal incontinence, urinary urgency, and decreased quality of life [5]. A study focused on the relationship between women with IBS and PFD reported their population was more likely to suffer from pelvic organ prolapse and urinary incontinence with constipation compared to their controls [5]. Few studies have been conducted linking the two conditions together and further strengthening the role of PFD in the pathogenesis of IBS-C is needed.

## Review

Methods

A comprehensive literature search was performed utilizing PubMed and Google Scholar. The key terms used to search for these articles were “irritable bowel syndrome”, “osteopathic manipulative medicine”, “OMM”, “pelvic floor dysfunction", “autonomic regulation”, and "enteric nervous system”. Articles were identified and analyzed by authors for eligibility between September 1, 2022, and February 1, 2023. Articles that were not published in English or were published before 2003 were excluded. Additionally, osteopathic manipulative medicine textbooks were consulted to obtain relevant treatment techniques and principles. No statistical analysis was performed nor was a Preferred Reporting Items for Systematic Reviews and Meta-Analyses (PRISMA) chart utilized for the literature review.

Results

**Figure 2:**
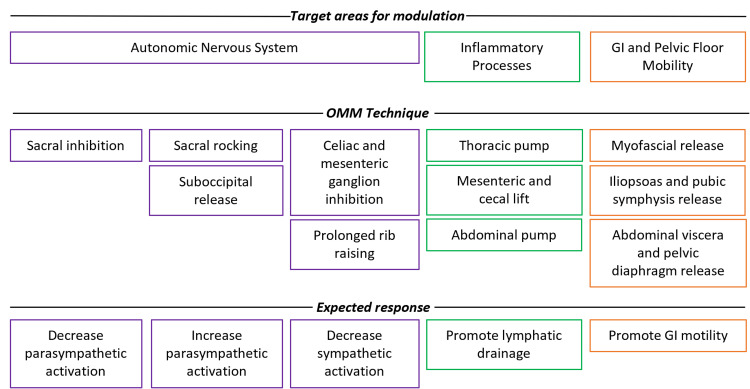
Schematic of proposed OMM techniques and their expected outcomes for the treatment of IBS Combined osteopathic manipulation medicine (OMM) induced modulation of the autonomic nervous system, inflammatory response mechanism, and gastrointestinal (GI) motility have predictable outcomes that would elicit biochemical responses that could improve symptoms of irritable bowel syndrome (IBS) in patients. Treatment techniques and their expected outcomes are displayed in columns and separated based on their target area of modulation. Original artwork by the authors.

Through its holistic approach, OMM may be able to restore balance within the ANS, reduce inflammation, reduce PFD, and improve symptoms and quality of life for patients with IBS-C. Pilot studies have shown that osteopathic treatment targeting the abdominal viscera may improve chronic constipation, the hallmark symptom of IBS-C [21]. Techniques that specifically target the ANS and promote homeostasis within the system may help restore the balance between the SANS and PANS. Stimulation of the PANS should promote increased gastrointestinal motility and relief of symptoms for IBS-C patients. Additionally, modulation of inflammation and addressing PFD provides alternative therapeutics. These treatments are highlighted in Figure 2.

Modulating inflammation

Myofascial Release (Biomechanical Model)

Fascia is a thin layer of connective tissue that envelops every organ, bone, nerve, blood vessel, and muscle in the body [22]. The intimate connection between fascia and various structures in the body means that any strain in the fascia can result in the compression of nerves, vessels, and lymphatics, which may impair their normal function [22]. Myofascial release (MFR) is used in a variety of different health conditions to treat myofascial restrictions and pain. In the context of treating IBS-C, MFR can be used to treat somatic dysfunction throughout the body to improve overall function. MFR techniques that target the craniocervical, thoracolumbar, iliopsoas, and abdomen may have a direct impact on the nerves, vessels, and lymphatics in these regions, promoting improved function of these systems. This approach is consistent with the third tenet of OMM, which emphasizes the reciprocal relationship between structure and function.

MFR applies pressure to fascial fibroblasts through indirect strain, and studies have shown that fibroblasts respond to mechanical loading in ways that depend on the magnitude, duration, and frequency of the strain. A study by Meltzer et al. using in vitro modeling showed that MFR treatment after repetitive strain injury resulted in a normalization of the apoptotic rate and a decrease in the production of inflammatory cytokines [23]. Although MFR is widely used, the quality and results of the research supporting its effectiveness are inconsistent [24]. Newly published studies using MFR to treat several different pain syndromes and vascular insufficiencies have laid a solid foundation for future research into the effectiveness of MFR [25-29]. Myofascial techniques include craniocervical spine release, thoracolumbar release, rib cage release, iliopsoas muscle release, and abdominal and viscera release.

Lymphatic (Respiratory-Circulation Model)

The lymphatic system is a vital component of the circulatory system that helps maintain homeostatic function throughout the body [30]. It serves several important functions, including regulating osmotic balance between the extracellular, intracellular, and intravascular fluid as well as removal and delivery of particles such as proteins, leukocytes, lymphocytes, and inflammatory mediators [30]. In the treatment of IBS-C, lymphatic techniques such as doming of the diaphragm, mesenteric lift of the cecum and small intestine, thoracic pump, pedal pump, abdominal pump, and rib raising can help improve lymphatic flow and aid in treating the pathological processes associated with IBS-C. During the inflammatory process, increased viscosity and stasis within venules and capillaries can occur due to the extravasation of fluid and plasma proteins from the intravascular space into the interstitial space [29]. In this context, the lymphatic system plays a critical role in promoting fluid drainage from inflamed tissues, and studies have shown that it can also help remove vasoactive mediators such as histamine, bradykinin, and prostaglandin E from sites of inflammation [30]. Removal of these vasoactive substances may directly impact increased mucosal permeability, which is a prominent pathological process in IBS-C [30]. Additionally, lymphatic drainage can help reduce inflammation by increasing the removal of macrophages from tissues [30]. Lymphatic techniques include doming the diaphragm, mesenteric lift of the cecum, mesenteric lift of the small intestine, thoracic pump, pedal pump, abdominal pump, and rib raising.

Autonomic nervous system targets

Parasympathetic Stimulation

Suboccipital release/Cranial Base Release/OA release: The suboccipital release is an OMM technique that is used to treat headaches and neck pain, and address autonomic dysfunction [31,32]. Compression of suboccipital muscles may lead to constriction of the vertebral arteries and suboccipital nerves, resulting in the clinical presentation of neck pain and headaches. In the suboccipital region, the VN exits the skull via the jugular foramen [33]. The myodural bridge represents a connection between the anatomical musculature and the central nervous system’s dura [32]. This technique has been shown to produce a direct stimulatory effect on the VN. The OA release has been shown to produce a direct stimulatory effect on the VN and thus resulting in increased parasympathetic tone. Implications of increased parasympathetic tone can influence colonic motor cells and IBS symptomatology depending on subtype. In IBS-C, due to the constipation-predominant symptoms, the increased parasympathetic tone may be useful in increasing peristaltic movements to regulate bowel movements.

Sympathetic Inhibition

Celiac, Superior Mesenteric, and Inferior Mesenteric Ganglion Inhibition: The gastrointestinal tract has sympathetic innervations that differ from the rest of the thoracic viscera. Preganglionic fibers arise from T9-L2 and pass through sacral, lumbar, and thoracic sympathetic splanchnic nerve branches to meet the prevertebral sympathetic ganglia on the abdominal aorta [34]. The sympathetic ganglia are divided into Celiac, Superior Mesenteric, and Inferior Mesenteric ganglia. These divisions parallel the embryological division of the gut into the foregut, midgut, and hindgut. Neurons within each ganglia attach to postganglionic fibers that provide innervation to abdominal and pelvic visceral tissue [34]. Axons innervate different regions of abdominal viscera by attaching to abdominal/pelvic vascular bundles that are connected to their target organs [34]. 

Modulation of sympathetic chain ganglia helps treat segmental facilitation in certain segments. Direct techniques are more commonly used when treating acute segmental facilitation by engaging the restrictive barrier. Indirect techniques can be used as well in patients whose visceral tissues are sensitive. Thus, physicians may use sympathetic ganglia inhibition to reduce IBS-C symptomatology, such as constipation by modulating the sympathetic nervous system.

Bilateral T10-L2 Paraspinal Inhibition

Sympathetic innervation to the head is provided by T1-T4 cell bodies. Somatic dysfunction elsewhere in the body may increase sympathetic tone in the head. Thus, hypersympathetic stimulation may provide physiologic visceral changes in the innervated tissue. These changes can manifest as Tissue texture changes, Asymmetry, Restriction of motion and Tenderness (TART) palpatory changes [33]. The palpatory changes found in the paraspinal location should prompt the physician to consider sympathetic innervation of organs at that level [35]. Through sympathetic nervous system inhibition, IBS-C symptomology can be reduced and treated.

Chapman Reflexes

Chapman Reflexes (CR) are thought to be caused by visceral dysfunction such as inflammation, spasm, and distention, which causes lymph stasis, thus resulting in congestion within the fascia [36,37]. When treating IBS-C patients, osteopathic physicians should monitor for anterior CR of the stomach, duodenum, pancreas, small intestines, colon, and liver, as well as organs that participate in the elimination of waste products such as the kidneys, spleen, and colon [36]. If any of these CRs are found, they should be treated with rotary stimulation for 20-60 seconds.

Sacral and Pelvic Dysfunction Treatments

In PFD treatment, there are a multitude of different modalities from lifestyle modifications, medications, and physical manipulation. Depending on the etiology, the treatment can be tailored to the patient. Current physical therapy interventions for hypertonic pelvic floor muscles are myofascial release, strain-counterstrain, and biofeedback [5]. The techniques that target the musculoskeletal component and function to relax the hypertonic muscles have been shown to improve patients’ urinary symptoms [5]. Hypertonic pelvic floor musculature has been investigated as an underlying etiology of dysfunction in the genitourinary and gastrointestinal tracts. The pelvic diaphragm forms part of the abdominopelvic cavity and thus has a role in the formation of the external anal sphincter. Treatment of the pelvic diaphragm can improve lymphatic circulation in the abdominal cavity. Taut fascia can lead to compression of lymphatic fluid and impede its circulation [24].

OMM can induce relaxation of overactive pelvic floor musculature and presents as a non-invasive treatment alternative for targeting constipation in IBS-C patients. Pubic symphysis, pelvic diaphragm, and sacroiliac releases are MFR techniques used to target taut tissues and musculature and induce relaxation. Manipulating the fascial restrictions directly targets the somatic dysfunctions such as hypertonic pelvic floor musculature and alleviates the strain on associated systems such as the genitourinary and gastrointestinal tract. The utilization of these techniques may lead to decreased reporting and severity of the symptoms caused by the underlying pathophysiology of PFD such as urinary incontinence and constipation.

Sacral rocking is an osteopathic technique primarily used to treat sacral dysfunctions; however, it is also indicated in dysfunctions characterized by decreased PANS activity and symptoms related to visceral organs. The target of sacral rocking is the PANS innervation from the spinal levels S2-S4 that supply the descending colon and the rectum [12]. Due to the connection of the sacrum and the innervation to the lower gastrointestinal tract, it is hypothesized sacral somatic dysfunctions can disrupt the innervation and lead to potential gastrointestinal dysfunction.

Such dysfunctions where sacral rocking can be indicated are for disorders dominated by constipation [21]. One of the main symptoms patients suffer from with IBS is constipation, and this could partly be due to the underlying decreased PANS activity leading to decreased secretions and peristalsis of the gastrointestinal tract. Sacral and pelvic dysfunction techniques include sacroiliac release, bilateral sacroiliac joint decompression, sacral rocking, pubic symphysis release, and pelvic diaphragm release.

Discussion

IBS-C stands as a persistent inflammatory gastrointestinal disorder that predominantly impacts the large intestine, manifesting with distressing symptoms including abdominal pain, cramping, gas, bloating, and constipation. The intricate tapestry of IBS-C pathogenesis encompasses multiple key elements, namely ANS dysfunction, immune-mediated inflammation, disruptions in the GBA, and microbiome dysbiosis. The gut microbiome exerts a profound influence on a multitude of biological processes through its pivotal role in the GBA, which in turn plays a critical role in regulating the Hypothalamic-Pituitary-Adrenal (HPA) axis, ENS, and the immune system. When the delicate equilibrium of the microbiome is disrupted, it triggers changes in gut motility and secretion, directly impacting the functionality of the gastrointestinal tract and thereby contributing to various conditions, including IBS-C.

Numerous contributing factors come into play in the complex web of IBS-C pathogenesis, encompassing stress, anxiety, dietary choices, and genetic predisposition. Pharmacologic therapy, such as antispasmodic, is often utilized in conjunction with gastrointestinal motility treatments to aid patients in symptom management. However, the disease burden differs vastly between patients with a range of mild to severe symptoms. Current non-pharmacological strategies for managing IBS-C primarily revolve around dietary modifications, exercise regimens, and psychological interventions. Osteopathic medicine, often overlooked in the realm of conventional medical approaches, stands as a holistic and comprehensive avenue for addressing IBS-C. Due to the philosophy of osteopathic medicine of viewing the patient as a whole, various treatments can be used to target specific symptoms that patients experience. Focusing on autonomic system modulation, control of inflammation, and pelvic and sacral floor dysfunction, the avenues for treatment combinations are multitudinous as seen in Figure 1.

## Conclusions

The prevalence of IBS has continued to rise, thus warranting alternative, non-pharmacologic therapeutic avenues to be explored. OMM poses a unique and valuable adjunct to traditional evidence-based medicine. This review aims to highlight the importance of future clinical trials, utilizing OMM as an additional treatment to improve the lifestyle of IBS patients. Future research endeavors should consider exploring treatment options for IBS-C to tailor OMM treatments based on the patient’s symptomatology.
